# Electrospun poly-4-hydroxybuterate scaffolds enable local zero-order estradiol delivery and promote collagen maturation in a hypoestrogenic rat model for pelvic organ prolapse repair

**DOI:** 10.1016/j.bbiosy.2026.100133

**Published:** 2026-02-28

**Authors:** Anel Oosthuysen, Carmen Weitsz, Jan‐Paul WR Roovers, Jaco Theron, Zeliha Guler

**Affiliations:** a Cardiovascular Research Unit, Christiaan Barnard Division of Cardiothoracic Surgery, University of Cape Town (UCT), Cape Town 7925, South Africa; b Department of Obstetrics and Gynecology, Amsterdam UMC, location AMC, University of Amsterdam, Meibergdreef 9, 1105 AZ Amsterdam, The Netherlands; c Amsterdam Reproduction and Development (AR&D) Research Institute, Amsterdam UMC, Meibergdreef 9, 1105 AZ, Amsterdam, The Netherlands

**Keywords:** Pelvic organ prolapse (POP), Controlled estradiol release, Collagen remodeling, Tissue regeneration, Degradable electropsun scaffolds

## Abstract

Pelvic organ prolapse (POP) affects millions of women globally, with postmenopausal hypoestrogenism playing a critical role in its development. Pelvic floor repair faces two major challenges: high recurrence rates and clinical complications that have been associated with permanent impants. Clinical complications may be reduced by using degradable implants. In fact, our previous research showed that absorbable poly-4-hydroxybutyrate (P4HB) implants result in fewer complications. We hypothesized that the high failure rates stem from impaired tissue regeneration and healing capacity, particularly in postmenopausal women requiring prolapse surgery who experience hypoestrogenism. Since estrogen is essential for tissue regeneration and pelvic floor integrity, we developed electrospun (ES) P4HB scaffolds with controlled estradiol (E2) release to enhance healing at the surgical site. In this study, we aimed to improve tissue regeneration through controlled release of estradiol (E2) at the surgical site. We investigated electrospun (ES) P4HB scaffolds loaded with E2 as a biodegradable alternative for POP repair. P4HB ES scaffolds with varying E2 concentrations (0%, 1%, 2%, and 5%) were fabricated and characterized for their physicochemical, mechanical, degradation, and drug eluting properties. Scaffolds had a suitable pore structure for tissue ingrowth and were strong and elastic enough to comply with native vaginal tissue, even after *in vitro* degradation for 20 weeks. *In vitro* drug release followed zero-order kinetics, with sustained E2 elution over 19 to 110 days. To evaluate *in vivo* host response, scaffolds were implanted subcutaneously in an ovariectomized rat model simulating postmenopausal estrogen deficiency. Both ES P4HB and ES P4HB-E2 scaffolds exhibited excellent biocompatibility, with no infections observed. Notably, ES P4HB-E2 scaffolds demonstrated significantly higher collagen type I/III ratio (11.5±6.3 *vs* 7.3±3.3, p=0.047), indicating enhanced collagen maturation and tissue remodeling. Total collagen deposition was high with no fibrotic response. LC-MS analyses confirmed local E2 delivery without systemic effects. The controlled local E2 release offers a promising therapeutic approach for POP treatment in hypoestrogenic patients.

## Highlights

• Both ES P4HB and ES P4HB-E2 scaffolds demonstrated sustained and controlled estradiol release, providing consistent hormonal support throughout the healing period.
• The scaffold architecture was specifically designed to support tissue integration and cell infiltration, promoting effective regeneration and wound healing.
• The mechanical properties of both ES P4HB and ES P4HB-E2 scaffolds closely matched those of native tissue, ensuring appropriate biomechanical support during the healing process.
• ES P4HB-E2 scaffolds demonstrated improved collagen metabolism, contributing to superior tissue remodeling and regeneration in the hypoestrogenic environment.

## Graphical abstract

**Figure:**
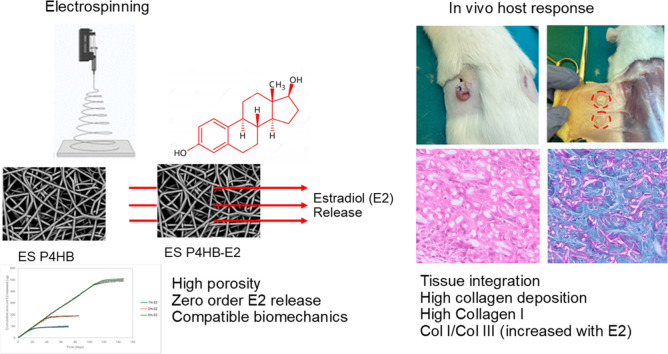

## 1 Introduction

POP is a debilitating condition characterized by the descent of pelvic organs into the vaginal cavity due to weakening of the pelvic floor muscles and connective tissues [1,2]. This condition affects approximately 1 in 4 women worldwide, with established risk factors including vaginal childbirth, advancing age, and hypoestrogenism, particularly in postmenopausal women [[3], [4], [5]]. POP may result in urinary or defecatory dysfunction, interference with sexual activity, and vaginal bleeding [1,2]. The lifetime risk for surgical intervention due to POP ranges from 6% to 19%, representing a substantial healthcare burden that is projected to increase significantly as populations age [6,7]. Native tissue repair, which uses patients' tissue for mechanical support, remains the primary surgical approach. However, the recurrence rates remain high due to the altered and impaired connective tissue quality that causes the low tensile strength of the vaginal wall [8]. As a result, the use of synthetic mesh reinforcements is prompted. However, nonabsorbable polypropylene (PP) meshes have been associated with severe complications including mesh erosion, chronic pain, dyspareunia, and organ perforation [9,10], leading to FDA warnings and market withdrawals [11]. These challenges have created an urgent need for alternative biomaterials that can provide temporary structural support while allowing native tissue regeneration. The Scientific Committee on Emerging and Newly Identified Health Risks (SCENIHR) by European Commission recommended to focus on safety of the new implants by using degradable implants and on the promotion of tissue regeneration [12].

Researchers have explored numerous biomaterials as alternatives to polypropylene meshes for pelvic organ prolapse (POP) treatment, each with unique advantages and limitations. Rapidly degrading materials like polyglactin-910, which absorbs completely within 6 weeks, and methoxy polyethylene glycol-polylactic-co-glycolic acid polymers have shown high recurrence rates (up to 25%) due to insufficient load-bearing capacity during tissue healing [[13], [14], [15]]. Xenografts such as InteXEN® [16] and Surgisis® [17] offer low erosion rates but similarly suffer from premature degradation. In contrast, non-degradable polypropylene meshes provide long-term support but cause complications including erosion, exposure, and chronic inflammation. Bacterial cellulose (BC) has emerged as a promising candidate, combining tensile strength comparable to steel with muscle-like softness and a collagen-resembling nano-fibril architecture that triggers only mild inflammatory responses [18]. BC meshes demonstrate mechanical properties equal to or exceeding polypropylene while achieving excellent tissue integration—in ewe vaginal implantation studies, surrounding tissue returned to normal muscle physiology after 90 days without severe inflammation. Collagen-coated BC (BCCOL) enhances cell adhesion in vitro but induces chronic inflammation in vivo, likely from glutaraldehyde residues [19]. Other innovative approaches include knitted polyamide meshes delivering mesenchymal stem cells and tropoelastin:PCL yarns showing minimal pro-inflammatory response [20]. In our group, we have been investigating Poly(4-hydroxybutyrate) (P4HB) as a long-term resorbable materials to reduce or eliminate the adverse events [[21], [22], [23], [24], [25], [26], [27]]. P4HB distinguishes itself through optimal degradation kinetics—degrading gradually while maintaining structural integrity during the critical tissue remodeling phase, thus providing sustained mechanical support without the long-term complications of non-degradable materials or the premature failure of rapidly degrading alternatives. Our previous work, reported mild host response with less adverse events and improved vaginal tissue biomechanics with knit P4HB meshes as compared to permanent mesh [24,28]. To improve tissue–scaffold interactions, we engineered electrospun P4HB scaffolds with an extracellular matrix (ECM)-mimicking architecture, providing a high surface-to-volume ratio and high porosity that favor scaffold–cell interactions and a constructive host response [22]. We demonstrated superior cell proliferation and collagen and elastin deposition compared to knitted designs, highlighting the advantages of this fabrication approach and promotion of the fibroblast behaviour on electrospun scaffolds (Supplementary document S1).

In the current study, we aimed to enhance tissue regeneration through controlled release of E2 at the surgical site. Drug delivery systems offer controlled, targeted release of therapeutic agents to enhance treatment efficacy while minimizing side effects. While extensively studied in other medical fields such as in cancer treatment through i.e nanoparticle-based systems [29] or in wound healing via stimuli-responsive materials like silk fibroin [30], drug delivery for prolapse treatment remains nascent compared to other medical applications, due to the challenges such as lack of standardization in outcome measurement and the slower pace of device evaluation compared to pharmaceuticals [31]. Nevertheless, integrating drug delivery with tissue engineering represents a promising frontier for improving prolapse treatment outcomes [32]. Previous approaches include PLA meshes with ascorbic acid derivatives for collagen stimulation [33], polyurethane scaffolds releasing 17-β-estradiol for enhanced integration in ex ovo chick chorioallantoic membrane (CAM) assay [34], PCL-based implants with growth factors and stem cells [35,36] and a nano-hydroxyapatite reinforced PCL mesh delivering metronidazole, ketorolac, bleomycin, and estradiol demonstrated sustained release for over 28 days in vivo [37]. Electropsun-based implants represents a very promising solution to the challenges in POP repair.

The current research represents a significant advancement by developing an estradiol-delivering poly(4-hydroxybutyrate) (P4HB) electrospun scaffold specifically for pelvic organ prolapse treatment. P4HB offers superior biocompatibility and mechanical properties compared to previously used polymers, while the estradiol incorporation addresses the hormonal deficiency underlying prolapse pathophysiology. We developed bioabsorbable E2-loaded ES P4HB scaffolds (ES P4HB-E2) designed to serve as both a mechanical support and a controlled E2 delivery system for POP repair. The incorporation of E2 into ES P4HB scaffolds addresses another critical factor in POP pathophysiology and treatment. Hypoestrogenism in postmenopausal women, who are major patient population, impairs wound healing, collagen synthesis, and tissue regeneration, contributing to both POP development and poor surgical outcomes [38,39]. Local vaginal estrogen administration has been shown to restore vaginal architecture, enhance angiogenesis, reduce inflammation, and promote ECM production, yet systemic administration carries risks of cardiovascular and neoplastic complications [40,41]. Controlled local delivery of E2 directly at the surgical site offers the potential to maximize therapeutic benefits while minimizing systemic exposure and related adverse side-effects.

We characterized the microstructure, mechanical properties, degradation kinetics, and E2 release profiles of the ES P4HB and E2-loaded (ES P4HB-E2) scaffolds *in vitro*.Subsequently, we evaluated the scaffolds in a hypoestrogenic rat model that mimics the postmenopausal state, assessing systemic E2 uptake, tissue integration, inflammatory response, vascularization, and overall wound healing. Our findings demonstrate that ES P4HB-E2 scaffolds successfully provide sustained local E2 release, enhance tissue regeneration with very minimal inflammation, promote collagen metabolism, and support robust tissue ingrowth during the critical healing period. These results suggest that ES P4HB-E2 scaffolds represent a promising bioabsorbable and hormonally enhanced alternative for surgical management of POP in postmenopausal women.

## 2 Materials and methods

### 2.1 Materials

Poly-4-hydroxybutyrate (P4HB) was obtained from Tepha Inc., Cambridge, MA and 17β- estradiol (E2) from Sigma Aldrich/Merck. Solvents chloroform and N,N-dimethylformamide (DMF), were of analytical grade and dried on molecular sieve before use.

### 2.2 Electrospinning of ES P4HB and ES P4HB-E2 scaffolds

Solutions containing 10wt% P4HB in a mixture of chloroform and DMF (9:1 v/v) were prepared. E2 was incorporated at concentrations of 0, 1, 2, or 5wt% relative to the polymer, resulting in homogenous solutions and producing scaffolds referred to as ES P4HB, ES P4HB-E2(1%), ES P4HB-E2(2%) and ES P4HB-E2(5%), respectively.

In a custom-built electrospinning rig, two high-voltage power supplies (ES60P-20W/CIC2 and ES30N-20W, Gamma High Voltage Research, USA) were used to create an electric field between a negatively charged (-2.2kV) rotating mandrel (diameter 25mm, rotation speed 1000rpm) and a positively charged (+17.8kV) spinneret (blunt hypodermic needle, 18G). Polymer solution was dispensed by a syringe pump (Chemyx Fusion 100, Stafford, USA) at a flow rate of 5ml/h via the spinneret and collected as a fibrous scaffold on the mandrel. The distance between the needle and mandrel was 300mm, and humidity was controlled at 35%. Scaffolds were placed in a vacuum chamber overnight to remove any residual solvent. Scaffolds designated for *in vivo* experiments were sterilized using a cold cycle ethylene oxide process [42].

### 2.3 Scaffold characterization

#### 2.3.1 Morphology and microarchitecture

##### 2.3.1.1 Image acquisition

Scanning electron microscopy (SEM) (JSM-IT200 InTouchScope™, JEOL Ltd., Japan) was used to examine the scaffold surface morphology. Images were acquired under low vacuum with a landing voltage of 10 kV and a magnification of 500 × .

##### 2.3.1.2 Fiber diameter, orientation and pore size

Fiber diameter and orientation, and pore size (n=6) were determined from SEM images using open source image processing software Fiji [43] with DiameterJ and OrientationJ plugins. Measured pore dimensions were converted to its equivalent circular diameters. Orientation of fibers was expressed as a coherency value (C) between 0 and 1, with 1 being aligned fibers and 0 random orientated fibers.

##### 2.3.1.3 Porosity

Porosity, defined as the ratio of void volume to the total volume of the scaffold, was determined by a liquid displacement method based on Archimedes’ principle. Samples (10mm diameter discs, n=6) were weighed in air and then in heptane as auxiliary liquid on an analytical balance equipped with a density determination kit (METTLER TOLEDO® Balance XS105 DualRange, Microsep (Pty) Lyd, SA).

$$\text{Porosity}=1-\frac{v_{f}}{v_{T}\;}=\;1-4\cdot \frac{m_{air}-m_{liq}}{\pi d^{2}t\left(\rho_{iq}-\rho_{air}\right)}$$

where *V_f_* and *V_T_* represent the bulk fiber and total sample volume, m_air_ and m_liq_ are the sample masses in air and heptane, ρ is the density of the liquid, and d and t are the diameter and thickness of the sample. The total sample volume was calculated based on the measured sample dimensions.

#### 2.3.2 Hydrodynamic properties

##### 2.3.2.1 Surface wettability

The surface wettability was determined by measuring the static water contact angle (q_C_) using the sessile drop method. Droplets of DI water (5µl) were gently deposited onto ES scaffolds and smooth solvent cast P4HB surfaces (n=5), and the droplets were imaged 3 seconds later using a digital microscope (TECHGEAR EagleScope, China). Contact angles were measured using the Fiji plugin ‘Contact Angle’.

##### 2.3.2.2 Water absorption

Water absorption by ES scaffolds and solvent cast films was monitored over time by immersing dry, pre-weighed (W_0_, 5-decimal analytical balance METTLER TOLEDO) discs (10mm diameter, n=6) in phosphate buffered saline (PBS) at 37°C. Samples were removed at pre-determined time points, patted dry, and weighed again (W*_t_*) until equilibrium was observed. Results were plotted as a ratio of W_t_/W_0_ *vs* time.

#### 2.3.3 Mechanical properties

##### 2.3.3.1 Uniaxial tensile testing

Dogbone-shaped samples were cut from ES scaffolds (gauge length 22mm, width 4.75mm, thickness varied between 0.52 and 0.84mm (individual sample thickness were used to convert force (N) to stress (MPa) for each sample), oriented either circumferentially or longitudinally relative to the mandrel axis during electrospinning. Uniaxial tensile tests were performed using a universal tensile tester (Instron 5544, Norwood, USA equipped with a 500N load cell). Samples (n=3) were stretched at an extension rate of 10mm/min until failure. Ultimate tensile strength (UTS) is the applied stress (force/sample cross-section area) at which the sample fails and eE_max_ is the strain (ΔL/L_0_) at which this happens, meaning elongation at break. The initial linear gradient (up to 5% strain) of the stress-strain curves was used to calculate the modulus.

##### 2.3.3.2 Suture retention

Samples (8 × 20mm, n=3) were cut from ES scaffolds in circumferential and longitudinal orientations. A suture (3/0 PP monofilament, CliniSut, South Africa) was passed through the sample 2mm from one end and tied in a loop with a double surgeon’s knot, while the opposite end of the sample was clamped in the bottom grip of a tensile tester (Instron 5544, Norwood, USA). The suture loop was hooked over a pin connected to the upper grip, which moved upwards at a crosshead speed of 10mm/min. The force required to tear the suture from the scaffold was recorded and normalized by the sample thickness and suture diameter, to give suture retention strength (SRS) expressed in MPa [44,45].

#### 2.3.4 *In vitro* hydrolytic degradation

The hydrolytic degradation rate of ES P4HB scaffolds in aqueous solution was assessed by monitoring the mass loss over time. The initial mass (m_0_) of each sample (10mmx10mm, n=6) was recorded before being pre-wetted in 20% EtOH and subsequently incubated in sealed tubes containing 1ml of neutral (physiological PBS, pH7.4), basic (0.05M NaOH, pH=13.7) or acidic (0.5M HCl, pH=0.3) solution at 37°C. The basic and acidic conditions represent accelerated in vitro degradation [46]. At pre-determined time points, samples were removed from the degradation solution, rinsed in DI water, dried, and weighed. The mass loss was calculated using the formula

$$mass\;loss\;\left(\%\right)=\left(1-\frac{m_{t}}{m_{0}}\right)\times \;100$$

The changes in morphology over the degradation period were also visualized by SEM to capture surface and structural changes. Mechanical properties and SRS of degraded scaffolds were measured to evaluate the functional impact of degradation over time.

#### 2.3.5 *In vitro* drug elution

The release profile of E2 from ES P4HB-E2 scaffolds was determined under *in vitro* conditions. Scaffolds with and without E2 (pre-weighed and pre-wetted, 10 × 10mm, n=3) were immersed in 1ml PBS in sealed tubes and incubated at 37°C with constant stirring. The eluates were removed daily and replaced with fresh PBS, thus maintaining sink conditions. The eluates were analyzed for E2 content using UV spectrometry (Shimadzu UV-1601PC spectrometer). Absorbance of each eluate was measured at λ=220nm and converted to E2 concentration (μg/ml) using a standard absorbance curve that was generated from E2/PBS standards. Cumulative release was calculated as the sum of E2 mass released at each time point according to:

$$Cumulative\;amount\;E2\;released\;\left(\mu g\right)=\sum_{i=1}^{t}C_{i}\times V$$

where $C_{i}$is the measured E2 concentration at time point *i* and *V* is the elution volume. The cumulative release of E2 was plotted as a function of time to evaluate the release kinetics and the potential for sustained drug delivery.

### 2.4 *In vivo* wound healing and tissue regeneration

#### 2.4.1 Ethics

**Fig. 5:**
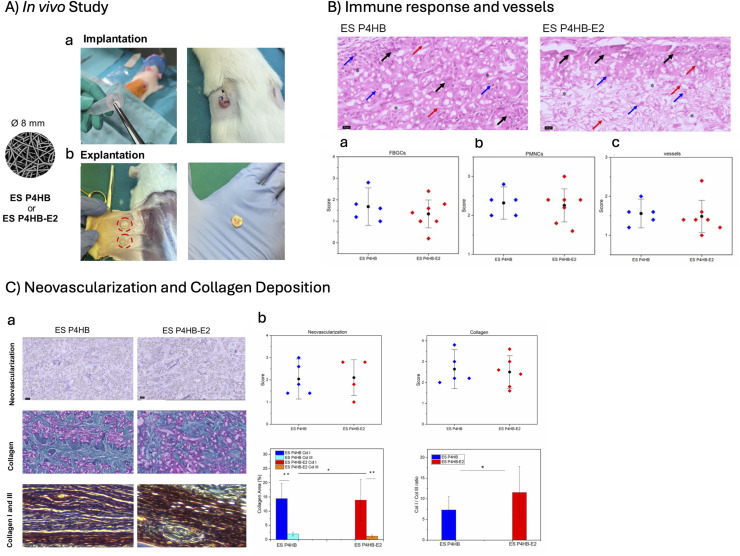
**A) In vivo study design showing s**ubcutaneous implantation of ES P4HB or ES P4HB-E2 scaffolds at the dorsum of the rat (a) and explanation images showing the healed tissue and top view of scaffold-tissue integration (b). B) Immune response and vessel presence evaluated after H&E staining: representative images of histomorphology of HE stains of ES P4HB and ES P4HB-E2 scaffolds (top) and mean scores for presence of a) FBGCs, b) PMNCs, and c) vessels (bottom). * = scaffold location, black arrow = FBGC, blue arrow = PMNC, red arrow = vessel. C) Neovascularization and collagen deposition evaluated after CD31 (neovascularization), Masson trichrome (collagen) and picrosirius red (polarized for Col I (red) and Col III (yellow)) stains of ES P4HB and ES P4HB-E2 scaffolds (a), and mean scores for neovascularization, collagen deposition, Col I and Col III area and Col I/Col III ratio presented (b). Error bars represent standard deviations (SD) or median ± interquartile range. Two-way ANOVA and Tukey's multiple comparisons test between individual groups were used to test for differences between groups. * Indicates p <0.05, ** indicates p < 0.001. All statistical results with p < 0.05 are written out in text in the manuscript.

In this study, a subcutaneous rat model was used to investigate the host response towards ES P4HB and ES P4HB-E2 scaffolds (Fig. 5A). The animal study protocol was approved by the University of Cape Town Animal Ethics Committee (Protocol nr 021_024). Experiments were performed in accordance with Animal Protection Act 1962 and South African National Standard for the care and use of animals for scientific purposes (SANS10386). In this study, only rats were used for investigation; patients were not included.

#### 2.4.2 Study design and sample size

10 Female Wistar rats (8-12 weeks old, weighing 200-300g) were ovariectomized (OVX) to induce iatrogenic menopause. Following a 14-day recovery period, the hypo-estrogenic status of the rats was confirmed by LC-MS analysis of blood plasma samples prior to scaffold implantation. The details of OVX can be found in the Supplementary document (S2). Rats were randomly implanted with ES P4HB and ES P4HB-E2 (2% E2) scaffolds (8mm diameter discs) subcutaneously at the dorsum. One or two longitudinal, para-vertebral incisions (1cm length) per rat were made, and a pouch was created at each incision site. A single scaffold was placed in each pouch in a random order and closed with 5/0 nylon suture, using an intradermal technique with a buried knot. All surgical procedures were performed under general inhalation anaesthesia. Randomization was performed by a computer-generated randomization list, random.org, Random Sequence Generator. All rats were acclimatized for at least 7 days before OVX surgery and were provided with sterile food and water *ad libitum.*

The sample size was calculated based on one-way ANOVA with a significance level of 10% and a power of 90% to observe a 20% difference in healing response (tissue ingrowth) between scaffold groups.

#### 2.4.3 Sample collection and outcomes

During the implant period, blood was drawn (0.75ml weekly, and 2ml upon euthanization) for the determination of systemic estrogen levels. Rats were euthanized by gas inhalation, followed by KCl injection at 14 days post-implantation for wound healing assessment. The scaffold-tissue complexes were harvested and cut into pieces for histology and immunohistochemistry.

##### 2.4.3.1 Histomorphology

Details of histology and immunohistochemistry (IHC) staining and scoring are provided in Supplementary document (S3). Histology sections were stained with haematoxylin and eosin (H&E) and Masson’s trichrome to quantify the foreign body giant cells (FBGC), polymorphonuclear cells (PMN), blood vessels and collagen deposition. Picrosirius red staining was performed to evaluate collagen fibers (Col I and Col III) under polarized microscopy. The Col I/Col III ratio was calculated by using Image J (S3). IHC staining was performed for the detection of neovascularisation (CD31). Semiquantitative assessment of all samples was performed by two individual researchers blinded for treatment groups, using a previously designed grading scale [24,28,47].

##### 2.4.3.2 Serum estradiol levels

E2 concentrations were measured in serum using the liquid chromatography-tandem mass spectrometry (LC-MS/MS) method of the Amsterdam UMC. E2 was extracted from serum using a hexane/ether (4:1 v/v) mixture. The supernatant was dried and reconstituted in methanol/water (1:1 v/v). E2 concentrations were measured with a 2D Xevo TQ-S mass spectrometer (Waters Corp., Milford, MA) with electrospray in negative mode and two Acquity UPLC analytical columns (C4 and C18, Waters Corp, 2.1 × 50 mm, 1.7μm particle size). Gradient elution was achieved using methanol/water mixtures (water containing 100 μM NH4F) at a flow rate of 0.6 and 0.4 ml/min. Transitions monitored were m/z 271 to 145 and 183, and m/z 274 to 148 and 186 for E2 and the internal standard, respectively. All samples were analysed in duplicate [48].

#### 2.4.4 Statistics

Statistical analyses for *in vitro* scaffold characterization were performed by JMP®, Version 18.2.2, SAS® Institute Inc., Cary NC, USA and Microsoft Excel® (Microsoft 365®, 2016). Statistical analyses of *in vivo* data were performed using Minitab19 software (Minitab, State College, PA, USA). Data normality and equal variance were tested by the Shapiro–Wilk and Levene`s tests, respectively. Two-way ANOVA was used for normally distributed data and multiple comparisons between individual groups using Tukey’s test. Non-normally distributed data were compared using the Mann–Whitney test, with additional Steel correction to adjust p-values for multiple comparisons. The data are reported as mean ± standard deviation (SD) and as a median with interquartile range (IQR), quartiles 1 and 3, for normal and non-normally distributed data, respectively. The significance level was defined as p < 0.05.

## 3 Results

### 3.1 Morphology and microarchitecture of the scaffolds

**Fig. 1:**
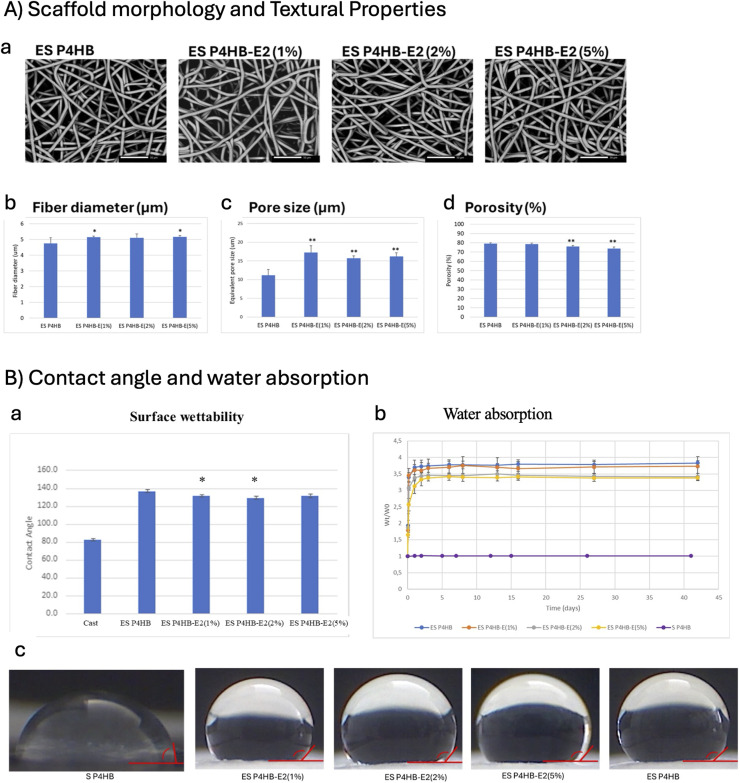
**A) Scaffold morphology and textural properties showing** SEM images of electrospun P4HB scaffolds (magnification 500x, scale bar 50µm) (a), fiber diameter (b), pore size (c) and porosity (d) of ES P4HB and ES P4HB-E2 scaffolds with increasing (1%, 2% and 5%) concentrations of estradiol (n=6, *p<0.05, **p<0.001). B)Water contact angle (a, (n=5), c) and water absorption (b, n=6) of the P4HB scaffolds (*p<0.05).

The morphology of the ES P4HB control and drug incorporated scaffolds with increasing concentrations of E2 (1%, 2% and 5%) was imaged with SEM (Fig. 1A-a). The ES P4HB scaffold showed a highly uniform, porous structure with continuous, smooth and defect-free fibers. These fibers were tangled and randomly oriented, mimicking the architecture of natural ECM. No fiber fusion was observed. Pores were of irregular shape and appeared interconnected. Upon incorporating E2 at 1%, 2%, and 5% concentrations, no discernible changes in fiber morphology of ES P4HB-E2 scaffolds were observed. The E2-containing fibers also appeared smooth, with no visible crystals on the surface, suggesting homogeneous incorporation of E2 into the polymer matrix. The average fiber diameter for ES P4HB scaffolds were 4.8±0.4μm (Fig. 1A-b). After incorporation of E2, the fiber diameter increased by 7.5 - 8.8% to 5.2±0.1μm (1%E2), 5.1±0.3μm (2%E2) and 5.2±0.1μm (5%E2), with only increases for 1%E2 and 5% E2 statistically significant (p<0.05). Coherency values for all scaffolds were below 0.1, indicating that there was no fiber alignment in a specific direction, but rather a totally random orientation distribution, rendering the scaffolds isotropic. Pore sizes increased significantly (p<0.001) from 11.2±1.6μm (ES P4HB) to 17.2±1.9μm, 15.7±0.6μm and 16.2±1.0μm after addition of 1, 2 and 5% E2 (Fig. 1A-c). Increasing E2 concentration significantly reduced scaffold porosity in a dose-dependent manner (79.2 ± 0.9% (ES P4HB) *vs* 78.6 ± 1.2% (ES P4HB-E2 (1%)), 76.1 ± 1.0% (ES P4HB-E2 (2%), p<0.001), 73.9 ± 1.6% (ES P4HB-E2 (5%), p<0.0001) (Fig. 1A-c).

### 3.2 Hydrodynamic properties of the scaffolds

Surface wettability, characterized by the water contact angle (WCA) of the scaffolds, was determined (Fig. 1B-a,c). P4HB, as a relatively hydrophobic material, exhibited a smooth surface WCA of 82.5 ± 1.4°, while the ES P4HB surface displayed a significantly higher contact angle of 136.7 ± 2.2° (Fig. 1B-a), reflecting the increased surface roughness of the ES scaffolds. Addition of E2 to the ES scaffolds led to a modest but significant reduction in contact angle for all E2-loaded groups (131.5±1.8° (ES P4HB-E2(1%)), 129.0±2.2° ES P4HB-E2(2%)), and 131.0±3.0° (ES P4HB-E2(5%)), (p<0.05 for 1 and 2%E2).

The water absorption behavior of the scaffolds was assessed to understand their interaction with the aqueous environment (Fig. 1B-b). Solid, non-porous films of P4HB displayed negligible mass changes over time, indicating minimal water uptake by the hydrophobic polymer itself. In contrast, porous ES scaffolds exhibited a gradual increase in mass as water infiltrated the void spaces, reaching equilibrium absorption after approximately four days. Scaffolds with higher E2 content exhibited reduced water absorption.

### 3.3 *In vitro* degradation of scaffolds

**Fig. 2:**
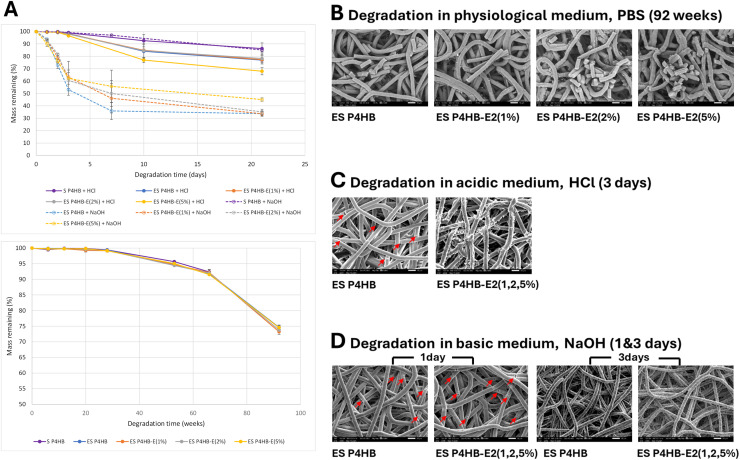
In vitro degradation of ES P4HB and ES P4HB-E2 scaffolds (A, n=6), and SEM images of the scaffolds degraded in PBS for 92 weeks (B), in HCl for 3 days (C), and in NaOH for 1 and 3 days (D). Red arrows indicate cracks. Image magnification 1000x.

*In vitro* degradation showed that mass loss due to long-term hydrolytic degradation of P4HB scaffolds in PBS (physiological pH 7.4) is slow, with over 70% mass remaining after 92 weeks (Fig. 2A). No significant differences were observed between porous ES scaffolds and solid cast films over this period, and the addition of E2 did not affect the degradation rate. Accelerated degradation of P4HB ES scaffolds and solid films in acidic (solid lines) and basic (dotted lines) media were compared (Fig. 2A). Solid films degraded at similar rates in both media, while ES scaffolds degraded faster in acid and even faster in basic medium. The high surface-to-volume ratio of ES scaffolds most likely promoted surface erosion in these environments.

**Fig. 4:**
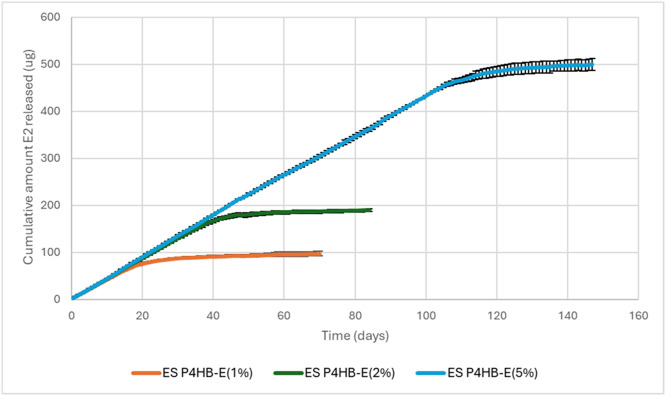
Cumulative estradiol released (μg) from 10mg P4HB scaffolds (n=3).

SEM images revealed fiber fragmentation of ES P4HB and ES P4HB-E2 scaffolds following degradation in PBS for 92 weeks (Fig. 2B), leading to the observed mass loss. Surface erosion is also visible on the ES P4HB-E2(5%) fibers. All E2 has been released from the scaffolds at this timepoint (Fig. 4). Acidic conditions (Fig. 2C) caused some fiber fracturing and slight surface erosion of P4HB after 3 days of exposure. E2 crystals were exposed from drug-containing fibers and aggregated on the fiber surfaces. Caustic NaOH caused all fibers (with and without E2) to fracture after only 1 day of exposure (see red arrows in Fig. 2D. Longer exposure time (3days) causes rough, eroded surfaces. No E2 crystals are observed on scaffolds, most likely because they dissolve more readily in NaOH (2.71mg/l @ pH10) than in HCl (1.48mg/l @ pH4) and PBS (1.51mg/l @ pH 7) [49].

### 3.4 Mechanical properties

**Fig. 3:**
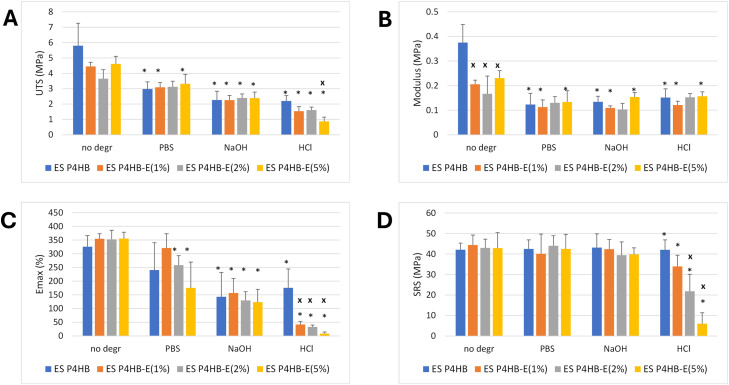
Mechanical properties of ES P4HB and ES P4HB-E2 scaffolds before and after in vitro degradation (n=3). * p<0.05 for comparison to corresponding non-degraded group, x p<0.05 for comparison to ES P4HB from same degradation treatment.

Fig. 3 shows mechanical and suture retention properties of scaffolds determined by tensile testing before and after *in vitro* hydrolytic degradation.

In accordance with the isotropic character of the scaffolds, no significant differences in mechanical behavior were observed between test results in circumferential and longitudinal orientations. Therefore, data from these groups were pooled. Prior to degradation, ES P4HB scaffolds demonstrated a UTS of 5.8±1.5MPa, Fig. 3A. The corresponding modulus, E_max_ and SRS values were 0.37±0.07MPa, 326.8±40.5% and 42.1±3.2MPa, respectively (Fig. 3B,C,D). Addition of E2 to the scaffolds nearly halved the stiffness (p<0.05, Fig. 3B), but did not significantly affect their tensile strength, E_max_ or SRS.

*In vitro* degradation for 20weeks in PBS (∼99% mass remaining) significantly weakened P4HB scaffolds (UTS=3.0±1.5MPa, p=0.022, Fig. 3A) and rendered them more elastic (0.12±0.05MPa, p=0.022, Fig. 3B), while the effects on E_max_ and SRS were significant (Fig. 3C,D) The effect of PBS degradation on mechanical properties was independent of the amount of E2 in the scaffolds.

Scaffolds were also degraded in basic and acidic media for accelerated results. The gross mass loss for neat P4HB scaffolds in 0.05M NaOH and 0.5M HCl were similar (∼90%) after respectively 1 day and 7 days, and the mechanical properties were comparable (Fig. 3). Increased addition of E2 did not noticeably affect base degradation. However, HCl-exposed scaffolds exhibited more pronounced reduction in E_max_ and SRS, with increasing E2 content. Polymer chain scission in acidic medium [50], together with the disruption of the molecular structure by E2 incorporation, may explain the reduced mechanical performance in these groups.

### 3.5 E2 release kinetics from ES P4HB-E2 scaffolds

Fig. 4 presents the cumulative release profile of E2 from P4HB scaffolds over time. No burst release was observed. Instead, all drug loadings followed the same linear release pattern, indicative of a zero-order release model. The majority of the incorporated E2 (76.2%, 86.7%, and 93.2% for 1%, 2%, and 5% E2 scaffolds, respectively) was released in a controlled, linear manner over 19, 39, and 110 days. Following this period, the release rate declined due to drug depletion. The complete release of E2 occurred over approximately 40 days for the 1% E2 scaffolds, 60 days for the 2% E2 scaffolds, and 145 days for the 5% E2 scaffolds.

### 3.6 *In vivo* host response and wound healing

Hypoestrogenic state of the animals were confirmed before the implantation. Before surgery E2 levels in rat blood serum varied between 5 and 112pmol/l, depending on the oestrus status. Following OVX the levels dropped to below 5pmol/l, and were maintained at these levels during the implant period. Host responses to ES P4HB and ES P4HB-E2 scaffolds at 2 weeks post-implantation were compared (Fig. 5). Both scaffolds exhibited good tissue integration without any signs of infections or exposures.

Both ES P4HB and ES P4HB-E2 scaffolds exhibited comparable and moderate immune response, evidenced by mild foreign body giant cell formation (1.7 ± 0.7 SD *vs* 1.3 ± 0.7 SD) (Fig. 5B-a) and moderate immune cell infiltration (2.3 ± 0.3 SD *vs* 2.3 ± 0.5) (Fig. 5B-b). There were no statistical differences in the presence of vessels (Fig. 5B-c) and neovascularization (Fig. 5C-b) between scaffolds. Masson trichrome and Picrosirius stains (Fig. 5C-a,b) confirmed collagen formation around and in the scaffolds at 2-weeks post-implantation. Collagen deposition around the ES P4HB and ES P4HB-E2 scaffolds was high (2.6 ± 0.8 SD vs 2.5 ± 0.7 SD), even though there was no statistical difference between the groups. Both scaffolds exhibited statistically significantly higher Col I than Col III fibers around the scaffolds (ES P4HB: Col I and Col III= 14.5 ± 5.4 SD and 2.0 ± 0.7 SD, p=0.00; ES P4HB-E2: Col I and Col III= 13.9 ± 7.4 SD and 1.2 ± 0.5 SD, p=0.00). There were no differences in Col I around both scaffolds, however Col III was statistically significantly (p=0.001) higher around the ES P4HB scaffolds. In addition, ES P4HB-E2 scaffold showed statistically significantly (p=0.047) higher Col I/Col III ratio as compared to ES P4HB (11.5 ± 6.3 SD *vs* 7.3 ±3.3 SD).

## 4 Discussion

Pelvic organ prolapse affects approximately 25% of women globally, with postmenopausal hypoestrogenism playing a critical role in its development and progression [3,26]. The hypoestrogenic state, characteristic of this patient population, poses significant challenges for surgical outcomes, as estrogen is essential for optimal wound healing, promoting vascularization, collagen synthesis, tissue strength, and reducing inflammation [39,51]. In this study, we investigated and compared the host response and tissue regeneration capacity of drug-free ES P4HB and E2-releasing (ES P4HB-E2) scaffolds in a subcutaneous rat model. Before *in vivo* implantation, both scaffold types were thoroughly characterized in terms of morphological, surface, mechanical, and degradation properties. The E2 release profile of ES P4HB-E2 scaffolds was established to guide concentration selection for the *in vivo* studies. ES P4HB-E2 scaffolds containing 2% E2 demonstrated sustained hormone release and significantly enhanced collagen maturation (higher Col I/Col III ratio) compared to scaffolds without E2, suggesting improved tissue remodeling for pelvic floor reconstruction.⁠

Successful pelvic floor repair requires scaffold materials that fulfill several critical criteria. The biological scaffold must exhibit good biocompatibility to facilitate cell adhesion and proliferation without toxicity or immunogenicity, be biodegradable to allow tissue ingrowth and replacement, possess adequate mechanical strength to guide tissue generation, and feature suitable porosity and pore size for vascularization. Our characterization of ES P4HB scaffolds demonstrated that E2 incorporation preserved the homogeneous fibrous architecture, with comparable fiber diameters observed between ES P4HB and ES P4HB-E2 scaffolds. The increase in fiber thickness, and accompanying increase in pore size and decrease in porosity, following incorporation of 1% E2, can be attributed to an increase in spinning solution viscosity [52], which supresses jet instability and limits jet stretching during electrospinning. Further increases in E2 concentration led to additional viscosity-driven fibre thickening, while reduced lateral jet motion resulted in more compact fibre deposition on the collector. As a result, denser fibre packing produced scaffolds with smaller pore dimensions and reduced porosity. Despite these microstructural changes, the intrinsically hydrophobic nature of ES P4HB scaffolds was only modestly altered by E2 incorporation, with a slight decrease in hydrophobicity and water absorption capacity observed. The homogenous incorporation of E2 into the scaffolds can be attributed to the lipophilic nature of E2 and its compatibility with the chloroform/DMF solvent system used during electrospinning. Similar findings have been reported after effective E2 incorporation into ES polymer fibers without morphological disruptions [34,53]. The porosity achieved in our scaffolds exceeded 70%, providing sufficient space for cells to migrate freely through the scaffold structure. Vaginal fibroblasts are able to penetrate ES scaffolds with pores between 3-8 μm in size [22,54]. The larger pores observed in our scaffolds may enable deeper cellular infiltration and enhanced collagen deposition, which are critical for successful tissue integration. Thinner fibers provide larger surface areas for protein absorption and cell binding, while appropriately sized pores facilitate cell migration and tissue integration. Additionally, randomly oriented fibers better replicate the structure of natural ECM compared to aligned fibers, promoting multidirectional cell adhesion and migration, which is a critical feature for applications involving dynamic mechanical forces such as POP repair. Our previous *in vitro* study [22] conducted with vaginal POP fibroblasts confirmed these favorable cellular interactions. ES P4HB scaffolds demonstrate tunable surface properties through fiber architecture and E2 incorporation. Surface wettability, measured by WCA, plays a key role in governing scaffold-cell interactions, affecting cell adhesion, proliferation, and migration [55]. ES P4HB scaffolds with and without E2 exhibited higher hydrophobicity relative to smooth P4HB surfaces, which is attributable to increased surface roughness [56] and the presence of air pockets within the fibrous architecture [57]. Notably, E2-containing scaffolds displayed lower WCA values despite thicker fibers, suggesting that P4HB and E2 interactions expose more hydrophilic end groups, reducing overall hydrophobicity. Water absorption studies revealed that P4HB does not absorb water or swell in an aqueous environment. Porous ES scaffolds gradually absorbed water into the void spaces until equilibrium was reached. Increasing E2 content, associated with both increase in hydrophobicity and lowering of scaffold porosity, restricted water infiltration and reduced equilibrium water absorption. The interplay between hydrophobicity, porosity, and water absorption can be utilized to regulate cell-scaffold interactions and drug release kinetics, rendering these scaffolds promising candidates for tissue engineering applications requiring sustained E2 delivery and favorable cellular responses.

Synthetic meshes for pelvic floor reconstruction must balance mechanical strength with tissue-like elasticity [24,28,58,59]. Current meshes often exhibit excessive stiffness compared to native tissue, contributing to complications. Tensile strength of ES P4HB scaffolds align with that of native vaginal tissue (0.27–6.4 MPa) [60]. E2 incorporation into P4HB scaffolds reduced tensile strength and stiffness, with lower modulus values likely due to the plasticizing effect that E2 has on the polymer structure. The lower stiffness region falls within the physiological range for tissue [61], making these ES scaffolds suitable for pelvic floor reconstruction. Suture retention strength showed no significant differences with the addition of E2 to the scaffolds. SRS is influenced by porosity [62] and fiber diameter [44], however, the variations observed in this study were too small to significantly influence the SRS of the scaffolds. SRS values of the scaffolds were found to be sufficient for a successful implantation as they exceed the suggested minimal SRS of 2.0 N [63,64].Overall, ES P4HB scaffolds demonstrated mechanical properties suitable for pelvic floor reconstruction, with E2 incorporation slightly reducing performance while maintaining clinical relevance for POP repair.

The release rate of E2 from the degradable, porous P4HB matrix was predominantly controlled by drug diffusion over the study period. Several factors influence this process, including the hydrophilicity and morphology of the matrix (e.g., fiber diameter and porosity), physicochemical properties of the drug (such as solubility and particle size), and interactions between the drug and scaffold material, such as hydrogen bonding between hydroxyl groups of E2 and polar carbonyl groups present in P4HB [65,66]. Consistent with diffusion-controlled release, no initial burst phase was observed. Burst release is undesirable due to potential local toxicity effects. Electrospun Polyurethane scaffolds releasing 17-β-estradiol with comparable pore size and lower fiber diameter to the ES P4HB and ES P4HB-E2 scaffolds were fabricated for the prolapse treatment [34]. However, they have reported initial burst release of 30-40% of E2 from electrospun PU scaffolds for 10 days. In our study, the absence of this phenomenon with the ES 4HB-E2 scaffolds suggests that E2 was uniformly distributed within the polymer matrix, with no free or weakly bound molecules on the scaffold surface. Given the low aqueous solubility of E2, particular care was taken to maintain sink conditions during the release study. Although the release volume was limited, complete daily replacement of the medium minimized saturation effects within each 24-hour interval. Interestingly, other groups who reported sustained E2 release without an initial burst phase also employed regular daily sampling protocols [66,67], whereas irregular or prolonged sampling intervals (>1 day) resulted in apparent burst release [34,53]. This discrepancy suggests that the low solubility of E2 may cause local saturation effects and sink conditions will no longer be maintained during longer sampling intervals, thereby restricting free E2 diffusion. Consequently, regular and frequent sampling is necessary to accurately mimic *in vivo* conditions, where drug metabolism occurs at a continuous rate. The observed release kinetics were independent of the initial drug loading, further supporting the observation of a zero-order release mechanism. This indicates that E2 release is primarily diffusion-controlled, with its inherently low solubility acting as a limiting factor. Therefore, the duration of drug release can be modulated by adjusting the initial drug loading. Given that P4HB exhibited minimal degradation in PBS (<0.75% mass loss over 20 weeks), polymer degradation or erosion likely had little to no impact on drug release within the measured timeframe, and the observed elution was predominantly driven by diffusion.

The investigated E2 loadings were fully released within 3–17 weeks, whereas the scaffolds retained more than 70% of their mass after 92 weeks of degradation. During this period, E2-loaded scaffolds exhibited average reductions of 25% in UTS and 29% in Emax, indicating some loss of mechanical integrity. Our previous *in vivo* sheep vaginal study [26], showed that both scaffolds with and without E2 improved the tissue biomechanics demonstrated by higher stiffness and ultimate strength compared to vaginal control tissue, despite the significant degradation (≈50%) of the scaffolds after 3-months post-implantation. Following implantation, the scaffold must initially maintain sufficient structural support to facilitate tissue ingrowth and progressive remodeling, thereby enabling the native tissue to gradually assume the load-bearing function as the synthetic support material degrades. The gradual and steady degradation process of the P4HB allows the surrounding tissue to remodel and achieve enough tensile strength to take over the mechanical load bearing of the scaffold. Fast degradation may result in recurrence of POP, therefore slow degradation of electrospun P4HB may be advantageous in the long term [26]. In contrast, estradiol delivery is primarily only required during the early post-implantation phase to enhance healing, tissue regeneration and modulate the initial inflammatory response. While the duration of estradiol application depends on on several factors including the type of surgery, patient age, and individual healing response, typically it is applied for 2-4 weeks post-operatively to support optimal wound healing [[68], [69], [70]].

Based on comprehensive characterization data, we selected the 2% E2-releasing scaffold for *in vivo* evaluation. This concentration demonstrated limited interference with scaffold properties while providing a therapeutically relevant amount of E2 release for up to 60 days. E2 was incorporated into the ES P4HB scaffolds as it is considered beneficial to multiple aspects of wound healing, including closure, neovascularization, and collagen synthesis. All E2 loadings characterized demonstrated limited impact on the scaffold properties and provided E2 at equal rates. We selected the scaffold with 2% E2 for *in vivo* evaluation, since it promised to provide E2 at a constant release rate for up to 39 days, far exceeding the implant period of 14 days. Although used for a different indication, low-dose E2 vaginal rings (ranging from 6.6 µg/day to 10 µg/day) have been found effective in reducing vaginal atrophy symptoms while minimizing systemic uptake [71].

Local release of E2 at the implantation sites contributed to wound healing, while analyses of serum E2 levels confirmed that there was no significant systemic uptake that could cause any adverse side effects.

Both ES P4HB and ES P4HB-E2 scaffolds demonstrated excellent biocompatibility throughout the implantation period, with no signs of infection or scaffold exposure observed. Electrospun scaffolds serves as platform for delivery to reduce complications. In a study of Laursen et al., the feasibility of using electrospun PCL scaffolds loaded with mesenchymal stromal cells and connective tissue growth factors for treating pelvic organ prolapse in elderly rats was conducted. Long-term studies using electrospun PCL meshes coated with connective tissue growth factor (CTGF)/PEG-fibrinogen and rat mesenchymal stem cells showed sustained mechanical support and biocompatibility up to 53 weeks in elderly rats, with reduced foreign body response and no implant-related complications [72]. Aside from delivery, degradable implants are considered to eliminate adverse events such as exposures. P4HB degrades slowly and gradually, which is ideal for proper tissue ingrowth and gradual load transfer. However, the timeframe of the current *in vivo* study evaluated host response after 2 weeks post-implantation, this timeframe did not provide sufficient time for significant P4HB degradation. Therefore, we can assume that scaffold degradation did not play a substantial role in the observed tissue response. Additionally, E2 release was not affected by scaffold degradation during this period. The ECM-mimicking structure of the scaffolds likely promoted tissue integration, resulting in good tissue-implant interaction.

Both scaffold types induced a moderate immune response, which can be explained by their high porosity allowing infiltration of immune cells. The incorporation of 2% E2 did not result in significant differences between scaffold groups in terms of immune response or neovascularization. Blood vessel formation and neovascularization around both scaffolds were moderate and comparable, likely due to the similar textural and mechanical properties of the scaffolds resulting in a comparable host response. Various polymer-based scaffolds have been investigated as alternatives to traditional polypropylene meshes for pelvic organ prolapse (POP) repair. Electrospun polycaprolactone (PCL) meshes modified with ureidopyrimidinone-motifs (UPy) have shown promising results in rat abdominal wall models, demonstrating physiologic musculofascial compliance and adequate mechanical properties [73]. In ovine models, electrospun meshes of ureidopyrimidinone-polycarbonate (UPy-PC) and polyurethane (PU) exhibited appropriate mechanical support, promoted neovascularization, and induced a milder inflammatory response with higher M2/M1 macrophage ratios compared to polypropylene [74]. Electrospun Polyurethane scaffolds releasing 17-β-estradiol with comparable pore size and lower fiber diameter to the ES P4HB and ES P4HB-E2 scaffolds were fabricated by Shafaat et al. [34]. They found E2 releasing PU scaffolds enhanced ECM production in adipose derived stem cells, and good cellular infiltration in CAM assay. However, comparison of the results of CAM assay and rat subcutaneous model is challenging due to the difference in experimental model. More advanced approaches have incorporated cellular components and growth factors. Bioprinted endometrial mesenchymal stem cells (eMSCs) encapsulated in Aloe Vera-Sodium Alginate hydrogel on 3D melt electrospun PCL meshes demonstrated enhanced tissue integration, cell retention, and anti-inflammatory M2 macrophage responses in NSG mice [75]. Compared to the complex multi-component systems involving growth factors, hydrogels, and stem cells, our E2-loaded P4HB scaffolds might offer a more streamlined and clinically translatable solution. The single-polymer system with incorporated hormone eliminates manufacturing complexity while directly targeting the pathophysiological mechanisms of postmenopausal POP.

It is known that ES implants tend to trigger foreign body response (FBR) more than knit implants due to their nonwoven and porous structure [76]. The high surface area-to-volume ratio and porosity of ES scaffolds mimic the natural ECM structure, providing an extensive and accessible fiber surface for cell and tissue interactions. This larger surface area allows for protein absorption and offers binding sites for cell membrane receptors. Consequently, fibroblasts integrate more effectively, achieving a more healing-oriented FBR [77].

Total collagen deposition around the ES P4HB scaffolds was high and did not show significant differences between groups. The higher collagen deposition might indicate mechanically stronger tissue. In POP, not only total collagen content but also the loose, discontinuous organization of collagen and the collagen type I to type III ratio are associated with disease progression [78]. Collagen, as the main component, accounts for up to 80% of connective tissue. Among the 28 types of collagen, types I and III are the most representative on the pelvic floor and are determinants of tissue strength [79]. Collagen type I has high stretching ability and resistance to tension, playing a crucial role in strengthening pelvic structures. Collagen type III provides flexibility and distension and is predominantly present in tissues subjected to periodic stress. Both collagen types I and III are found in tissue during wound healing [8,80]. Fibroblasts appear from early to late post-implantation and deposit collagen types I and III to repair damaged tissue [81]. However, implant properties such as porosity, construction type, degradability, and especially stiffness [82] can alter the host response and result in excessive collagen production, potentially leading to a fibrotic response [83]. Triggering proper healing without creating a fibrotic response is key in reconstructive POP surgery. In the case of ES P4HB scaffolds, there were no signs of fibrotic response.

During *in vivo* tissue remodeling, immature collagen type III is replaced by mature collagen type I, and deposition in the tissue increases over time. Both scaffold types exhibited higher collagen type I than type III content. Notably, collagen type I increased relative to collagen type III over time in both groups, indicative of tissue maturation. The increase in collagen type I after scaffold implantation can be attributed to the healing process and collagen metabolism. Importantly, ES P4HB-E2 scaffolds exhibited a significantly higher Col I/Col III ratio compared to ES P4HB scaffolds, suggesting enhanced collagen maturation and tissue remodeling in the presence of controlled E2 release. An increased collagen type I/III ratio contributes relevantly to the structural integrity of supportive tissue in the pelvic floor and could lead to enhanced remodeling, more optimal wound healing, and improved tissue strength [8,22,84].

### 4.1 Strengths and limitations

This study presents several notable strengths in its approach to evaluating E2-loaded P4HB scaffolds for POP treatment. The scaffolds were thoroughly characterized prior to the animal study, demonstrating zero-order release of E2 for up to 110 days, which to our knowledge represents a unique achievement in this field. This sustained release profile would be particularly beneficial for clinical applications in prolapse treatment, where long-term therapeutic effects are desired. The local delivery of E2 through the scaffolds showed meaningful effects on tissue healing response, particularly in triggering collagen maturation and enhancing tissue remodeling, as demonstrated by the significantly higher collagen type I/III ratio observed in ES P4HB-E2 scaffolds. The decision to evaluate a single E2 concentration was made consciously, adhering to the 3R principles to minimize animal use while still obtaining valuable preliminary data.

However, several limitations must be acknowledged when interpreting these findings. The subcutaneous rat model, while useful for evaluating biocompatibility and tissue response, has limited translatability to prolapse conditions due to differences in implantation location and the biomechanics involved in the disease process. The tissue response was assessed at only a single time point, whereas evaluation over a longer period could have provided insights into the scaffolds' effects across different phases of wound healing. The two-week duration of the *in vivo* study provided indications of early wound healing responses, but this timeframe may not have been sufficient to observe more dominant or pronounced systemic effects of the E2-loaded scaffolds. Given the limited but notable effects observed with local E2 delivery, further optimization of E2 concentration or a longer study duration would be beneficial to fully characterize the therapeutic potential of this approach and observe more substantial tissue remodeling outcomes.

## 5 Conclusions

This study successfully demonstrated that ES P4HB scaffolds with incorporated E2 offer a promising advancement in POP repair strategies. The scaffolds exhibited mechanical properties compatible with human vaginal tissue while enabling controlled, sustained E2 release following zero-order kinetics. Critically, the *in vivo* OVX rat model confirmed that locally delivered E2 remains confined to the target site without systemic circulation, thereby avoiding the potential adverse effects associated with systemic hormone therapy.

The 2% E2-loaded scaffolds significantly enhanced collagen maturation, as evidenced by elevated collagen type I/III ratios, indicating improved tissue remodeling crucial for durable surgical outcomes. The ECM-mimicking architecture of the ES P4HB scaffolds further promoted tissue integration and healing, with or without E2 incorporation. These findings, combined with the ability to customize drug loading and release duration, provide a versatile platform for tailored therapeutic interventions.

While these results are encouraging, comprehensive long-term preclinical and clinical studies are essential to fully establish the safety, efficacy, and durability of ES P4HB scaffolds in the treatment of POP. Nevertheless, this work, in conjunction with previous research, strongly supports the continued development of P4HB-based scaffolds as an innovative solution for localized, controlled estrogen delivery, with the potential to significantly improve patient outcomes in POP surgical repair.

## Funding

This work is supported by the Dutch Research Council (NWO) VENI Talent Scheme (17349) for the project ``Development of a novel wound healing implant with mechano-stimulation and localized delivery”.

## Declaration of generative AI and AI-assisted technologies in the manuscript preparation process

During the preparation of this work the author(s) used Notion AI, Notion Labs, Inc. (2025) in order to edit the text. After using this tool/service, the author(s) reviewed and edited the content as needed and take(s) full responsibility for the content of the published article.

## CRediT authorship contribution statement

**Anel Oosthuysen:** Writing – review & editing, Writing – original draft, Methodology, Formal analysis. **Carmen Weitsz:** Formal analysis. **Jan‐Paul WR Roovers:** Writing – review & editing, Resources, Investigation, Conceptualization. **Jaco Theron:** Writing – review & editing, Supervision. **Zeliha Guler:** Writing – review & editing, Writing – original draft, Supervision, Project administration, Methodology, Investigation, Resources, Funding acquisition, Formal analysis.

## Declaration of competing interest

The authors declare the following financial interests/personal relationships which may be considered as potential competing interests:

Zeliha Guler reports financial support was provided by Dutch Research Council. If there are other authors, they declare that they have no known competing financial interests or personal relationships that could have appeared to influence the work reported in this paper.

## Data Availability

Data will be made available on request.

## Appendix Supplementary materials

Image, application 1
