# Supplementary material for: Electrospun poly-4-hydroxybuterate scaffolds enable local zero-order estradiol delivery and promote collagen maturation in a hypoestrogenic rat model for pelvic organ prolapse repair
Source: Biomater Biosyst. 2026 Feb 28;21:100133. doi: 10.1016/j.bbiosy.2026.100133 (PMC12969649; doi:10.1016/j.bbiosy.2026.100133)
Supplement: Supplementary file 1 [file mmc1.docx]

**Supplementary**

**Electrospun Poly-4-Hydroxybuterate Scaffolds Enable Local Zero-order Estradiol Delivery and Promote Collagen Maturation in a Hypoestrogenic Rat Model for Pelvic Organ Prolapse Repair**

**Supplementary document 1 (S1)- *In vitro* data**

ES P4HB and ES P4HB-E2 scaffolds are shown to promote cell proliferation and collagen deposition of human vaginal fibroblasts[1].

Cell proliferation was assessed by Alamar blue colorimetric viability assay. Collagen deposition was imaged and determined following to Picrosirius Red (PSR) staining. The cell proliferation and viability, and collagen deposition by vaginal fibroblasts were increased over time.

**Cell proliferation**

At days 1, 7, 14, 21, and 28 vaginal fibroblast proliferation on scaffolds was assessed by a continuous Alamar blue colorimetric viability assay (Bio-Rad Laboratories, Inc. USA) based on metabolically active cells reducing resazurin (600 nm) to resorufin (570 nm). Three independent experiments were performed and per experiment three samples per scaffold type were evaluated. The scaffolds were transferred to a clean 24-well plate and incubated with 600 µl 10% Alamar Blue (10% v/v Alamar blue in supplemented DMEM) at 37 ºC with 5% CO2. After 3 h, the optical density (OD) in 150 µl of the Alamar blue solution was read trifold in a flat-bottom 96-well plate at 570 and 600 nm (SynergyTM H1 multimode microplate reader, Biotek Instruments Inc. USA). The average OD of three scaffolds was calculated after correction with the OD of a cell-free control mesh and the culture medium.

**Collagen deposition**

Collagen deposition was evaluated on days 14 and 28 using 0.1% Picrosirius Red (PSR) staining. After a 15-minute fixation in 4% paraformaldehyde the fixed samples were stained for 30 min in 400 µl PSR at room temperature. The stained scaffolds were imaged using a light microscope (Olympus BX41, Leica, Germany). In addition, collagen deposition was semi-quantitatively assessed by measuring the absorbance of the extracted dye at 540nm. For this, after staining, the samples were washed three times with 1 ml PBS and 1 ml of Extraction Buffer (Chrondrex, Inc., USA) was added. After resuspension, the absorbance was read trifold in 150 µl in a flat-bottom 96-well plate at 540 nm (SynergyTM H1 multimode microplate reader, Biotek Instruments Inc. USA). Using standard reference curves based on rat collagen I (Cultrex, R&D systems, USA), the amount of collagen was calculated in µg/scaffold. Three independent experiments were performed and per experiment three samples per scaffold type were evaluated.


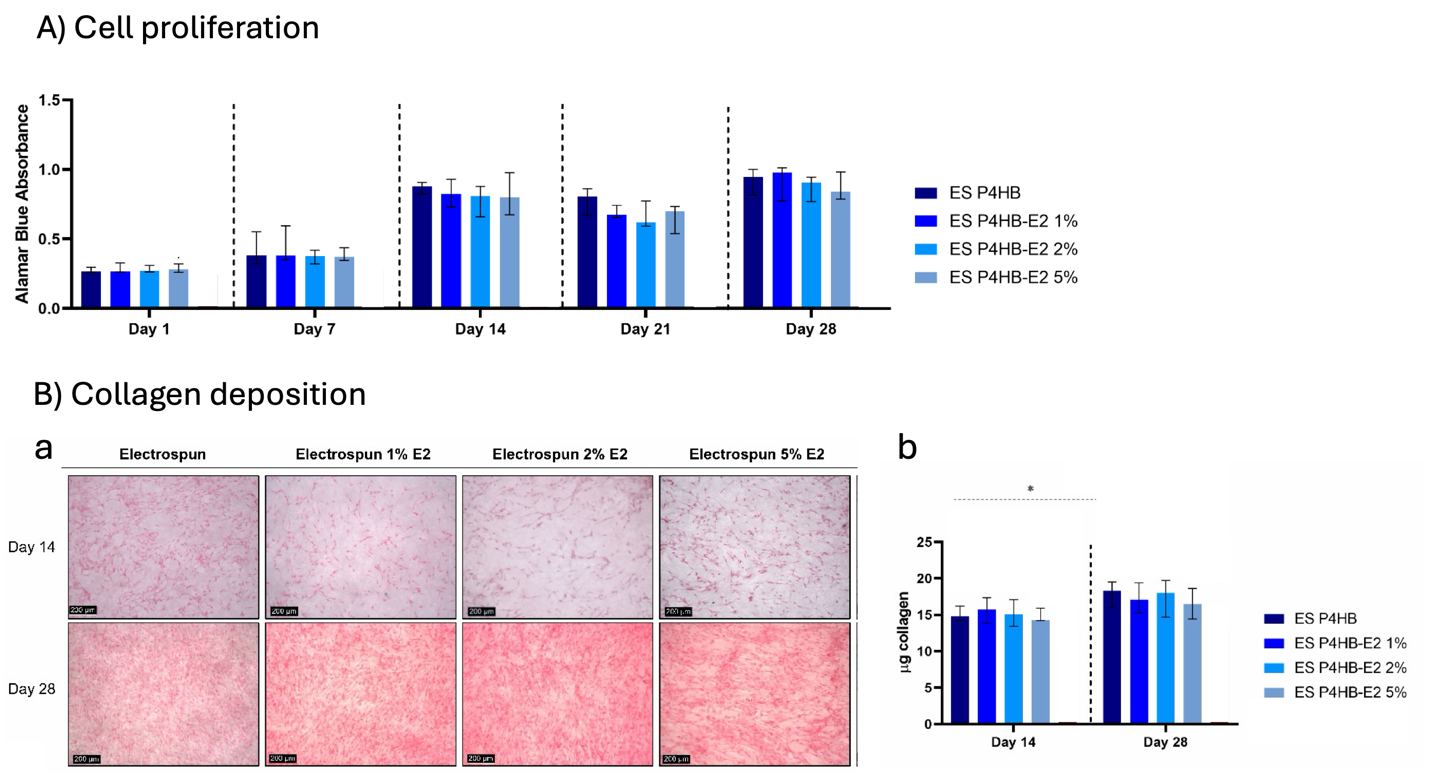


**Figure S1**. A) Cell proliferation was measured using an Alamar blue colorimetric viability assay on days 1, 7, 14, 21 and 28. B) Collagen deposition was imaged after a Picrosirius Red stain on days 14 and 28. a) Representative images demonstrate deposited collagen (red) on ES P4hB scaffolds and increased collagen deposition over time, and b) Collagen deposition increased over time, which reached significant differences (grey dashed line). Three independent experiments were performed and per experiment three samples per scaffold type were evaluated .* *p* < 0.05, ** *p* < 0.01 (images modified from previous publication[1].

**Supplementary document 2 (S2) - *In vivo* subcutaneous rat study**

***Housing and animal care***

Animals were obtained from the UCT Animal Unit at least seven days prior to surgery to allow for proper acclimatization, with both housing and the operating theatre located at the same facility. For anesthesia, animals were pre-medicated with buprenorphine (0.05mg/kg) administered 45-60 minutes before the procedure. Anesthetic induction was achieved using 5% isoflurane in oxygen for approximately three minutes in a chamber, followed by maintenance with 1.5% isoflurane via nose cone. Throughout the procedure, ocular lubrication was applied and body temperature was maintained at 37°C using appropriate warming equipment. Surgical plane of anesthesia was confirmed by loss of corneal and pedal response along with regular respiration. Post-operatively, animals were placed under a heat lamp and monitored until full consciousness and mobility were regained, then housed singly for the first 24 hours with welfare checks at 1, 4, and 6-8 hours after surgery, including daily monitoring and weighing thereafter. All surgical procedures followed strict aseptic techniques in a dedicated operating area using sterile instruments and materials, with the surgical site properly clipped and disinfected according to aseptic principles using warmed F10 skin prep solutions and/or betadine.

***Overiectomisation (OVX)***

The ovariectomy (OVX) procedure as a bilateral surgical intervention was performed on approximately 10-week-old rats under general anaesthesia using strict aseptic technique. The animal was positioned in lateral recumbency, and a 1 cm flank incision was made midway between the iliac crest and last rib, just cranial to the fourth abdominal mammary papilla. Through sharp and blunt dissection, the subcutaneous tissue and two muscle layers were carefully incised to access the peritoneal cavity. The ovarian fat pad was exteriorized, and the ovary was identified within it. Two ligatures using 5/0 Polyglactin 960 were placed around the proper ligament of the ovary and surrounding vasculature to ensure haemostasis. The ovary was then excised cranial to the ligature and removed. After confirming no bleeding from the uterine stump, the tissue was returned to the abdomen. The muscle layers were closed with simple interrupted or cross mattress sutures, and the skin was closed using intradermal sutures or clips to minimize wound interference. The procedure was repeated on the contralateral side. Post-operatively, the surgical site was cleaned and dried, the animal was placed in a clean recovery cage with appropriate heating, and 2ml of sterile saline was administered subcutaneously. Animals were monitored continuously until ambulatory, and post-operative analgesia was provided if pain was evident 6-12 hours after surgery.

***Subcutaneous implantation***

The surgical procedure was performed following a minimum interval of 14 days after ovariectomy (OVX). Longitudinal, para-vertebral incisions, each measuring 1 cm in length, were made using a scalpel blade, with three incisions created on each side of the spine. Through blunt dissection, a pouch with a depth of 2 cm was prepared at each incision site. A single scaffold was placed randomly into each pouch. The incisions were then closed using a 5/0 nylon suture with an intradermal technique and a buried knot. Just prior to recovery from anesthesia, a subcutaneous injection of 2 ml of sterile saline was administered to each animal. Post-operative analgesia was provided to animals that experienced pain 6-12 hours after the surgical procedure.

***Blood sampling***

Blood sampling was conducted by placing the rat in a restraint device with the tail extended. A heat lamp was positioned over the tail for 2-5 minutes to warm it and dilate the vessels. A 23G needle attached to a syringe was then inserted into a lateral vein, approximately 2-3 cm from the tip of the tail, and blood samples were collected (0.75 ml on a weekly basis or 2 ml when rats were sacrificed).

***Euthanasia***

Euthanasia was performed at defined time points using an overdose of an inhalation anaesthetic agent followed by CO2 exposure for 6 minutes. In cases where an animal suffered from a severe injury or infection with no perspective of recovery or with significant reduction of welfare, euthanasia was performed after consultation with a veterinarian. Death was confirmed by an intra-cardial injection of 1 ml saturated potassium-chloride solution.

**Supplementary document 3 (S3) - Histology and immunohistochemistry (IHC)**

Staining was performed on 5 µm sections and scoring of histology and immunohistochemistry (IHC) slides were performed by two researchers blinded to the groups on five randomly chosen non- overlapping fields per slide (5 samples per group) were scored at a magnification of ×400 and averaged (n=5). Fields were randomly selected at the interface between the scaffold and surrounding tissue. In case of any disagreement on scoring, a senior researcher was consulted for a third opinion.

Hematoxylin and Eosin (H&E) stains were performed to quantify the presence of foreign body giant cells (FBGC), polymorphonuclear (PMN) and vessels. An ordinal scale was used similar to that described in our previous studies [2-4], where scores are made as follows: none of the cells/vessels per high-power field (score 0-none), 1–5 (score 1-mild), 6–10 (score 2-moderate) and >10 (score 3-severe). Masson’s trichrome stains extracellular connective tissue (mainly unspecified collagen) blue. Five non-overlapping images 400x magnification were obtained and semi-quantitatively evaluated for collagen at interface scaffold-surrounding tissue. Samples were scored from 1-4 (Score 1: mild presence, Score 2: large presence, Score 3: abundance and Score 4: great abundance). Picro sirious images were analyzed via polarized microscopy, and the areas for collagen I and collagen fibers were analyzed via Image J. Polarized images were split into the red and green colour channels. The areas (%) of Col I fibers (visible as red) and Col III fibers (visible as yellow) were measured in red and green channels, respectively.

For IHC staining (CD31), sections were processed with a mouse and rabbit specific HRP/DAB (ABC) Detection IHC Kit (Abcam) and three different monoclonal antibodies (Table S1). Antibodies were diluted in 1% bovine albumin serum (BSA) (Sigma-Aldrich). Semi-quantitative assessment of the extent of immunostaining was performed using a qualitative grading scale; absent=0, mild presence=1, large presence=2, abundance=3, great abundance=4. Example photographs (Figure S1) depicting 1, 2, 3 and 4 were provided for reference.


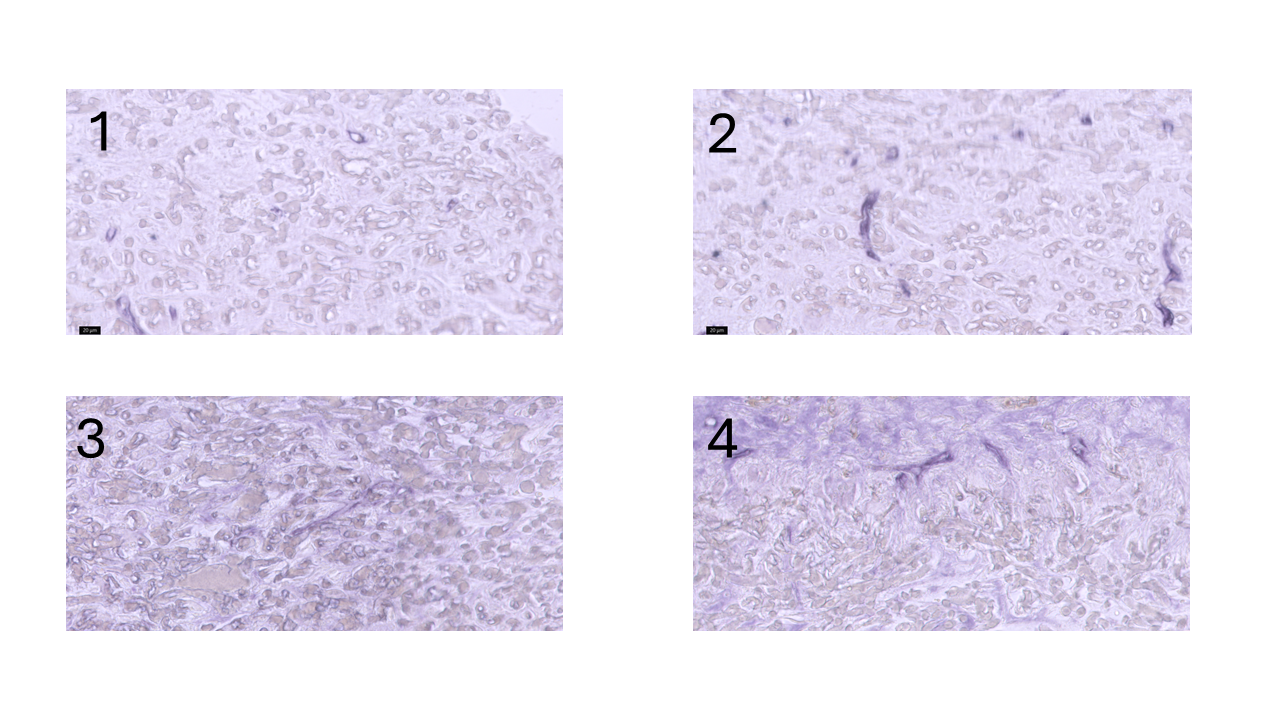


Figure S2: Example photographs depicting score 1, 2, 3 and 4 of CD31 stains were provided for reference.

**References**

1. Verhorstert, K., et al., *Absorbable Electrospun Poly-4-hydroxybutyrate Scaffolds as a Potential Solution for Pelvic Organ Prolapse Surgery.* ACS Applied Bio Materials, 2022. **5**(11): p. 5270-5280.

2. Gudde, A.N., et al., *Injectable polyisocyanide hydrogel as healing supplement for connective tissue regeneration in an abdominal wound model.* Biomaterials, 2023. **302**: p. 122337.

3. Guler, Z., et al., *Two-Year Preclinical Evaluation of Long-Term Absorbable Poly-4-hydroxybutyrate Scaffold for Surgical Correction of Pelvic Organ Prolapse.* International Urogynecology Journal, 2024. **35**(3): p. 713-722.

4. Diedrich, C.M., et al., *Evaluation of the short term host response and biomechanics of an absorbable poly‐4‐hydroxybutyrate scaffold in a sheep model following vaginal implantation.* BJOG: An International Journal of Obstetrics & Gynaecology, 2021.
